# Drought is threatening plant growth and soil nutrients of grassland ecosystems: A meta‐analysis

**DOI:** 10.1002/ece3.10092

**Published:** 2023-05-24

**Authors:** Cheng Liu, Muji Siri, Hui Li, Cheng Ren, Jing Huang, Changliang Feng, Kesi Liu

**Affiliations:** ^1^ College of Grassland Science and Technology China Agricultural University Beijing China; ^2^ National Field Station of Grassland Ecosystem in Guyuan Guyuan China; ^3^ Key Laboratory of Restoration Ecology of Cold Area in Qinghai Province, Northwest Institute of Plateau Biology Chinese Academy of Sciences Xining China

**Keywords:** drought, grassland ecosystem, microbial biomass, plant traits, soil nutrient

## Abstract

As a widespread direct effect of global warming, drought is currently wreaking havoc on terrestrial ecosystems' structure and function, however, the synthesized analysis is lacked to explore the general rules between drought changes and main functional factors of grassland ecosystems. In this work, meta‐analysis was used to examine the impacts of drought on grassland ecosystems in recent decades. According to the results, drought greatly reduced aboveground biomass (AGB), aboveground net primary production (ANPP), height, belowground biomass (BGB), belowground net primary production (BNPP), microbial biomass nitrogen (MBN), microbial biomass carbon (MBC) and soil respiration (SR), and increased dissolved organic carbon (DOC), total nitrogen (TN), total phosphorus (TP), nitrate nitrogen (NO3^−^‐N), and the ratio of microbial biomass carbon and nitrogen (MBC/MBN). The drought‐related environmental factor mean annual temperature (MAT) was negatively correlated with AGB, height, ANPP, BNPP, MBC, and MBN, however, mean annual precipitation (MAP) had positive effect on these variables. These findings indicate that drought is threatening the biotic environment of grassland ecosystem, and the positive steps should be taken to address the negative effects of drought on grassland ecosystems due to climate change.

## Graphical abstract

Drought led to significant decrease in AGB, BGB, ANPP, BNPP, MBC, MBN, and SR. However, some soil nutrients (DOC, TN, NO3^−^‐N, and TP) had significant increase under drought stress. Drought is threatening the biotic environment of grassland ecosystem, and the positive steps should be taken to address the negative effects of drought on grassland ecosystems due to climate change.

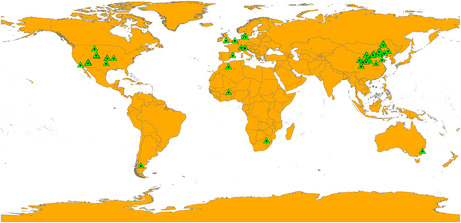

## 1 INTRODUCTION

Grassland, the largest terrestrial environment on Earth, accounting for roughly 40% of the global terrestrial landscape (O'Mara, 2012), has the potential to play a key role in greenhouse gas mitigation, particularly in global carbon sequestration (Soussana et al., 2004). Given that grasslands offer a variety of global goods supply and natural benefits, it is essential to evaluate the dynamic change in the grassland environment numerically (Gang et al., 2016; Zhou, Yang, et al., 2018), particularly under current climate change conditions. According to most of climate change forecasts, a large portion of the world's land area is expected to experience growing aridity (He & Dijkstra, 2014). Previous research has found that grassland ecosystems are more vulnerable to droughts than other ecosystems (Li et al., 2018; Wang et al., 2011), since most of grasslands reside in arid region. According to Liu, Zhu, et al. (2021), different types of grassland experience loss of functions due to drought, such as the decrease in productivity, water, and nutrient availability. A lack of water and diminished grass nutrients affect the livestock's resistance to disease, ability to reproduce, and survive.

Plants are an essential part of the terrestrial environment, regulating water cycle, matter and energy transfers, as well as temperature and soil carbon balance. Numerous studies have demonstrated that variations in precipitation trends have been linked to changes in plant community variety, primary productivity, functional composition, the quantity and quality of soil carbon sequestration, and the terrestrial carbon cycle (Du et al., 2015; Hooker et al., 2008; Reynolds et al., 2015). Long‐term precipitation deficits decrease the accessible water in the root zone of vegetation, and influence development and even raising mortality of vegetation (Liu, Zhou, et al., 2021; Wang, Huang, et al., 2021). Furthermore, extreme drought affects biomass allocation, net primary production (NPP), and carbon storage in grasslands. For instance, a recent meta‐analysis examining how plants allocate their biomass in reaction to drought revealed that while stem, leaf, and reproductive mass decreases, root mass increases considerably during drought (Eziz et al., 2017); a 4‐year experimental drought treatment on alpine grassland found that drought did not impact total net primary production (NPP) but instead moved more net primary production to belowground (Liu, Mi, et al., 2018); and extreme drought decreases both above‐ and belowground carbon storage in a temperate grassland environment (Chen et al., 2020).

Soil microorganisms have also been identified as the most sensitive component to changes in soil water availability in grassland ecosystems (Chen et al., 2019), and their biomass and structures are altered by the levels of drought (Mackie et al., 2019). Such as, in xeric ecosystems, water additions can significantly increase microbial biomass (Manzoni et al., 2014). Additionally, microbial reactions to variations of soil moisture differ greatly among species or functional categories (Zhao et al., 2017), for instance, fungus may be better able to withstand water duress than bacteria (Zhou, Wang & Luo, 2018). During drought, soil microbial communities synthesize extracellular polysaccharides (Marchus et al., 2018) and store simple carbon (C)‐ and nitrogen (N)‐rich osmolytes. During rewetting periods, microbes release stored osmolytes and reactivate the mineralization of organic substrates accumulated in soil to release absorbable nutrients for plant growth (Schimel, 2018).

Drought not only alters soil microbial composition but also reduces plant carbon inputs due to plant early senescence in dry condition (Schaeffer et al., 2017), and then causes soil carbon loss. Nitrogen is an important nutrient that can affect decomposition rates and storage of carbon. Drought, like its effects on soil carbon dynamics, has an immediate and indirect influence on nitrogen dynamics (Hartmann et al., 2013). Drought reduces soil nitrogen cycling rates, because water availability regulates hydration for the microbial processes fixing and transforming nitrogen, and controls substrate diffusion, microbial, and plant access to nitrogen (Homyak et al., 2017). Soil carbon and nitrogen were found to have a specific binding connection (Morillas et al., 2015). A meta‐analysis demonstrated that under droughts, soil total nitrogen (TN) concentration increased in tandem with soil organic carbon (SOC) concentration, and dissolved organic nitrogen (DON) increased in tandem with dissolved organic carbon (DOC) (Deng et al., 2021), as nitrogen availability increased as a result of improved availability of labile soil carbon (Larsen et al., 2011).

Drought, as previously stated, is likely to increase physiological stress due to decreased soil water availability, impacts on plants, soil microbes, and soil nutrients, and thus may create an imbalance of carbon and nitrogen cycles in grassland ecosystems. However, most of studies focus on local regions and use few parameters to explain the drought effects (Gao et al., 2021; Maxwell et al., 2022; Sieve et al., 2021), the synthesized analysis should be investigated to attain the general rules between drought changes and the key functional elements of grassland ecosystems. Therefore, in this study, the meta‐analysis was used to investigate the responses of plant indicators, soil indexes, and soil microbial biomass to the drought around the global grassland ecosystems. We hypothesized that (1) drought had a severe negative impact on plants, soil microbes, and soil nutrients of grassland ecosystems; and (2) the determinants of drought factors, such as MAT, MAP etc., had differentiated effects on plant, microbial biomass, and soil nutrients.

## 2 MATERIALS AND METHODS

### 2.1 Data collection and extraction

**FIGURE 1:**
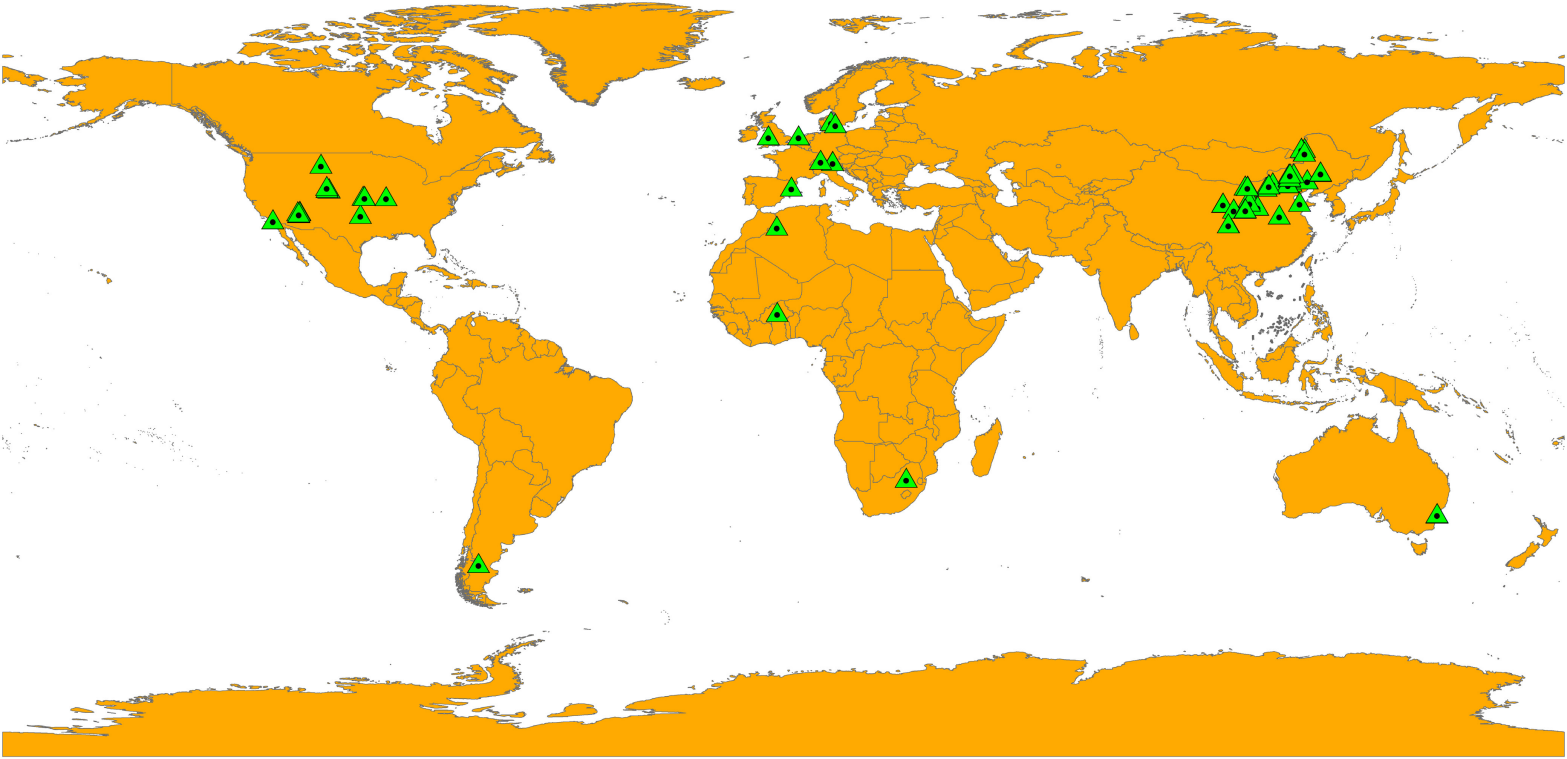
Geographic locations of the 80 studies, including 99 sites in this figure (green dot). Among them, 58 sites in China, 4 sites in Australia, 2 sites in Austria, 3 sites in Denmark, 17 sites in United States, 3 sites in United Kingdom, 2 sites in South Africa, 4 sites in Africa, 1 site in Spain, 3 sites in France, 1 site in Switzerland, and 1 site in Germany.

The Web of Science database (http://apps.webofknowledge.com) and the China National Knowledge Infrastructure (https://www.cnki.net/) were used to retrieve peer‐reviewed papers from 2000 to 2023. Several combinations of relevant keywords were used, including (grassland* OR steppe* OR rangeland* OR pasture* OR meadow* OR prairie*) AND (drought* OR drought experiment * OR precipitation exclusion * OR precipitation reduction* OR decreased precipitation* OR decreased rainfall* OR water stress* OR altered precipitation regimes*). The matching articles were found using the following criteria: (1) only field experiments in the grassland ecosystems were selected; (2) if the experiment included multiple treatments, only the data of drought treatment and control groups were selected; (3) except for the drought design differences, other factors in the drought and control treatment should be similar; (4) if various environmental variables were included in several trials within the same article, each experiment was classified as a separate research; (5) each chosen parameter should provide information on mean values, standard deviations (SDs) or standard errors (SEs), and sample size. In line with these criteria, 80 articles were selected from different study sites using GetData Graph Digitizer (version 2.24; http://www.getdata‐graph‐digitizer.com/; Figure 1). In addition, the Data sources section provides a list of the data sources utilized in the study (Appendix S1).

Within these articles, parameters related to the functions of grassland ecosystems under drought treatment were collected, including aboveground biomass (AGB), belowground biomass (BGB), height, aboveground net primary production (ANPP), belowground net primary production (BNPP), total nitrogen (TN), nitrate nitrogen (NO_3_^−^‐N), ammonium nitrogen (NH_4_^+^‐N), total organic carbon (TOC), dissolved organic carbon (DOC), total phosphorus (TP), available phosphorus (AP), microbial biomass nitrogen (MBN), microbial biomass carbon (MBC), ratio of microbial biomass carbon and nitrogen (MBC/MBN), and soil respiration (SR). Meanwhile, depending on latitude and longitude, the mean annual precipitation (MAP) and mean annual temperature (MAT) were retrieved from mentioned articles or acquired from the NOAA‐Climate Prediction Center (https://www.ncei.noaa.gov). Standardizing drought intensities between research by comparing the reduced rainfall in the experiment with MAP.

### 2.2 Statistical analysis

We used the natural log of the response ratio (RR) as the effective value to study the influence of therapy on the related parameters (Hedges et al., 1999). The RR was computed using the following formula (Gurevitch et al., 2001):

$$\mathrm{RR}=\ln\left(X_{t}/X_{c}\right)=\ln\left(X_{t}\right)-\ln\left(X_{c}\right)$$

where *X*_t_ and *X*_c_ are the corresponding mean values for the drought treatment and control groups.

To facilitate comprehension, the RR was converted to the percentage change = (e^RR^ − 1) × 100%.

In addition, the variance (*v*) of response ratio was computed:

$$v=\left(\frac{S_{t^{2}}}{n_{t}\times t^{2}}\right)+\left(\frac{S_{c^{2}}}{n_{c}\times c^{2}}\right)$$

where *S*_t_ and *S*_c_ represent the standard deviations in the drought treatments and the control groups, respectively, and *n*_t_ and *n*_c_ represent the sample sizes in the drought treatments and the control groups, respectively.

This mean effect size was determined using a random‐effects model (Gurevitch et al., 2013). We estimated the average response ratio (RR_*++*_) by summing the weights of the data pairs from all the investigations, using the formula below:

$${\mathrm{RR}}_{++}=\frac{\sum \left(\mathrm{RR}i\cdot w_{i}\right)}{\sum w_{i}}$$

where RR_*++*_ is the mean response ratio, RR_*i*_ is the weighted effective value of the *i*‐th data pair, and *w*_*i*_ is the pairwise weight. The following is how *w*_*i*_ was determined:

$$w_{i}=\frac{1}{V}$$

The 95% confidence interval of the comprehensive effect value indicated the variation of the effect value and was determined using the following formula:

$$95\%\mathrm{CI}={\mathrm{RR}}_{++}\pm 1.96S_{{\mathrm{RR}}_{++}}$$

where $S_{{\mathrm{RR}}_{++}}$ is the standard error of the means of response ratio, which was calculated as follows:

$$S_{{\mathrm{RR}}_{++}}=\frac{1}{\sqrt{\sum w_{i}}}$$

To better express the variation of indicators under the treatment group, the RR_++_ of indicator was converted to the percentage:

$$\;E=\left(\exp\left({\mathrm{RR}}_{++}\right)-1\right)\times 100\%$$

The relationship between parameters and drought‐related factors was analyzed using regression analysis based on the RR. The preceding studies and computations were performed with MetaWin 2.1 software, and GraphPad Prism 9.0 was used to plot figures (Systat Software Inc.).

## 3 RESULTS

### 3.1 Response of plant indicators, soil indexes, and soil microbial biomass to drought

**FIGURE 2:**
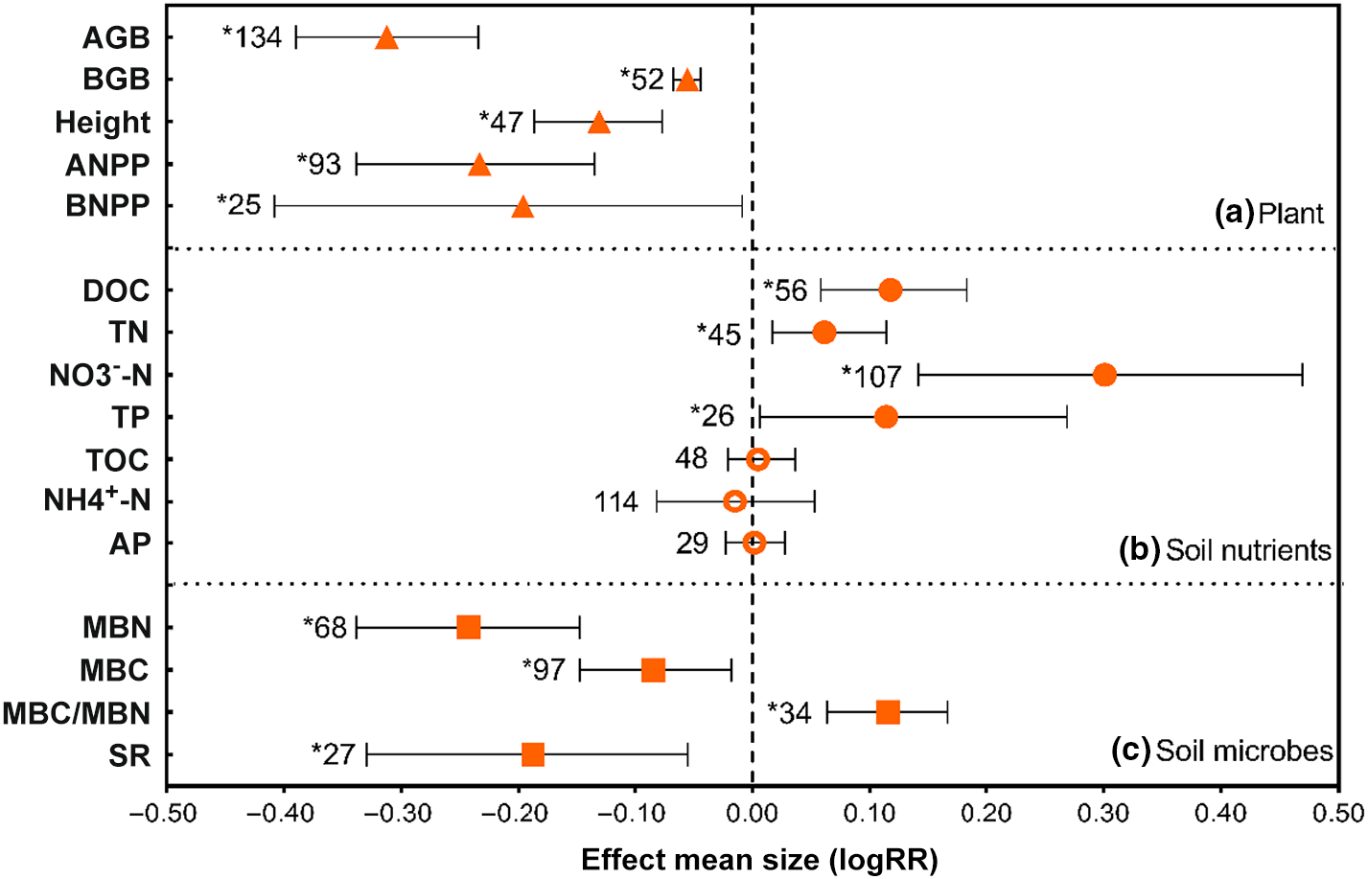
(a) The effect value of aboveground biomass (AGB), belowground biomass (BGB), height, aboveground net primary production (ANPP), and belowground net primary production (BNPP) as the reaction of drought. (b) The effect values of total organic carbon (TOC) dissolved organic carbon (DOC), total nitrogen (TN), nitrate nitrogen (NO_3_^−^‐N), ammonium nitrogen (NH_4_^+^‐N), total phosphorus (TP), and available phosphorus (AP) as the reaction of drought. (c) The effect values of microbial biomass carbon (MBC), microbial biomass nitrogen (MBN), the ratio of microbial biomass carbon and nitrogen (MBC/MBN), and soil respiration (Rs) as the reaction of drought. The black dashed lines show the log response ratio of 0.0, values are shown as the mean ± 95% confidence intervals of the percentage effects between drought treatments and control groups, and sample sizes are given to the graph. *Indicates significant effects of drought on variables.

Drought significantly decreased plant‐associated traits, including aboveground biomass (AGB), belowground biomass (BGB), height, aboveground net primary production (ANPP), and belowground net primary production (BNPP). Among them, the decrease level of AGB, ANPP, and BNPP was relatively high, and reached 31.22%, 23.33%, and 19.61%, respectively (Figure 2a). Soil main nutrients showed distinct fluctuation under drought stress (Figure 2b). Drought increased dissolved organic carbon (DOC) by 11.77%, total nitrogen (TN) by 6.14%, nitrate nitrogen (NO_3_^−^‐N) by 30.09% and total phosphorus (TP) by 11.39%, respectively (Figure 2b). Drought stress had no effect on total organic carbon (TOC), ammonium nitrogen (NH_4_^+^‐N), and available phosphorus (AP) (Figure 2b).

Microbial biomass generally decreased under drought stress (Figure 2c). Microbial biomass nitrogen (MBN) had relatively greater reduction compared to microbial biomass carbon (MBC), 24.21% versus 8.40%. This phenomenon caused the increase in the ratio of microbial biomass carbon and nitrogen (MBC/MBN) by 11.57% and the decrease in soil respiration (SR), and SR decreased up to 18.70% (Figure 2c).

### 3.2 Response of plant indicators, soil nutrient indexes, and soil microbial biomass to environmental factors and drought intensity

Environmental parameters such as mean annual temperature (MAT), mean annual precipitation (MAP), and altitude had varying influence on plant traits (Table 1). MAT had negative effect on AGB, height, ANPP, and BNPP. MAP had positive effect on these plant traits. Altitude had significant negative effect on AGB, BGB, and height. These plant traits except BGB had positive correlation with drought intensity (Table 1).

**TABLE 1:** Analysis of the logarithmic reactions of aboveground biomass (AGB), belowground biomass (BGB), height, aboveground net primary production (ANPP), and belowground net primary production (BNPP) under drought stress to mean annual temperature (MAT), mean annual precipitation (MAP), altitude, and drought intensity.

	AGB		BGB		Height		ANPP		BNPP	
	*m*	*p*/*n*	*m*	*p*/*n*	*m*	*p*/*n*	*m*	*p*/*n*	m	*p*/n
MAT	−	***/114	+	***/67	−	***/37	−	***/81	−	***/25
MAP	+	***/132	−	***/72	+	***/47	+	***/81	+	***/21
Altitude	−	***/51	−	***/29	−	***/20	+	***/34	−	0.098/3
Drought intensity	+	***/94	−	***/46	+	***/34	+	***/36	−	***/14

*Note*: + and − indicate slope direction (*m*). * means *p* < .05, ** means *p* < .01, *** means *p* < .001, *n* indicates sample size, and / reflects insufficient data.

Soil nutrient indexes had different correlation with MAT, MAP, altitude, and drought intensity (Table 2). Based on collected data, MAT had positive correlation with TOC, DOC, and NO_3_^−^‐N. However, MAP had negative correlation with DOC, NO_3_^−^‐N, NH_4_^+^‐N, TP, and AP, and positive correlation with TOC, TN. Altitude and drought intensity had similar positive effects on most of nutrient index (Table 2). As for microbes linked variables, MAT had negative effect on MBC and MBN. MAP had positive effect on MBC, MBN, MBC/MBN, and SR. Altitude had negative effect on MBC, MBN, MBC/MBN, and SR. Drought intensity caused positive effects on MBC, MBN, and MBC/MBN (Table 3).

**TABLE 2:** Analysis of the logarithmic reactions of total organic carbon (TOC), dissolved organic carbon (DOC), total nitrogen (TN), nitrate nitrogen (NO_3_^−^‐N), ammonium nitrogen (NH_4_^+^‐N), total phosphorus (TP), and available phosphorus (AP) under drought against mean annual temperature (MAT), mean annual precipitation (MAP), altitude, and drought intensity.

	TOC		DOC		TN		NO_3_^−^‐N		NH_4_^+^‐N		TP		AP	
	*m*	*p*/*n*	*m*	*p*/*n*	*m*	*p*/*n*	*m*	*p*/*n*	*m*	*p*/*n*	*m*	*p*/*n*	*m*	*p*/*n*
MAT	+	***/46	+	***/52	−	***/44	+	***/107	−	***/114	+	***/26	+	***/29
MAP	+	***/48	−	***/52	+	***/44	−	***/107	−	***/114	−	***/26	−	***/29
Altitude	−	***/33	+	***/44	−	***/33	−	***/65	+	***/72	−	***/19	+	***/16
Drought intensity	+	***/30	+	***/33	−	*/34	+	***/86	+	***/87	+	***/8	+	***/21

*Note*: + and ‐ indicate slope direction (*m*), * means *p* < .05, ** means *p* < .01, *** means *p* < .001, *n* indicates sample size, and / reflects insufficient data.

**TABLE 3:** Analysis of the logarithmic reactions of microbial biomass carbon (MBC), microbial biomass nitrogen (MBN), MBC/MBN, and soil respiration (SR) under drought against mean annual temperature (MAT), mean annual precipitation (MAP), altitude, and drought intensity.

	MBC		MBN		MBC/MBN		SR	
	*m*	*p*/*n*	*m*	*p*/*n*	*m*	*p*/*n*	*m*	*p*/*n*
MAT	−	***/97	−	***/68	+	***/34	+	***/27
MAP	+	***/97	+	***/68	+	***/34	+	***/27
Altitude	−	***/84	−	***/59	−	***/32	−	***/18
Drought intensity	+	***/45	+	***/33	+	0.521/34	−	***/17

*Note*: + and − indicate slope direction (*m*), * means *p* < .05, ** means *p* < .01, *** means *p* < .001, *n*indicates sample size, and / reflects insufficient data.

## 4 DISCUSSION

### 4.1 Drought effect on plant, soil microbes, and soil respiration

Drought is the most immediate effect of global climate change on terrestrial ecosystems (Craine et al., 2013). Therefore, drought probably caused significant effects on the components of grassland ecosystems, such as plant traits, soil nutrient properties, and soil microbes. Some findings from specific experimental sites have verified this conclusion (De Boeck et al., 2016; Fuchslueger et al., 2014; Knapp et al., 2015; Martorell et al., 2021). Synthesized the current global studies of drought stress on grassland ecosystems, we found several general phenomena associated with plant traits, soil nutrient properties, and soil microbes. Drought led to the significant decrease in AGB, BGB, plant height, ANPP, BNPP, MBC, MBN, and SR. The overall decrease in plant‐related traits could be attributed to drought‐induced plant physiological constraints and nutritional limitations (Meisser et al., 2019), since microbial N mineralization was hindered during the drought (Braun et al., 2018). Drought can also disrupt the soil microenvironment (Schroeder et al., 2016), decline plant root physiological (Liu, Konings, et al., 2021) and biochemical processes (Roca et al., 2015), weaken plant photosynthesis (Meeran et al., 2021), and subsequently result in decrease in productivity (Gould et al., 2016). The decrease in microbial activity, due to drought can prevent the mineralization of soil organic matter (Mganga et al., 2019), descend soil nutrient cycling (Wang et al., 2015), decline soil quality (Berdeni et al., 2021), and result in negative feedback in the “plant–soil–microbe” system (Li, Zhang, et al., 2020), and consequently decrease plant growth (Luo et al., 2020). Root respiration is a major contributor to SR, and changes in BNPP have a significant effect on soil respiration (Xu et al., 2015). SR is generally reduced by the increase in the biomass of microbes or plants (Zhang et al., 2019), which corresponded to the findings of plants and microbes in this study. Drought reduces soil water availability and inhibits plant growth, root respiration, and microbial activity, and then decreases soil respiration (Ru et al., 2018). MBC/MBN increased when drought occurred, which is consistent with the finding of Sun et al. (2020).

### 4.2 Drought effect on soil available nutrients

The decrease in plant growth and soil microbial biomass reduces their nutrient requirements from the soil, especially available nutrients (Dijkstra et al., 2015; Fay et al., 2015), then might increase the accumulation of DOC, TN, NO_3_^−^‐N, and TP in soil, which was consistent with the findings in this study. In water‐stressed environments, Liu, Lü, et al. (2018) revealed that plants transfer more carbon to the subsurface as a survival strategy to maximize their ability to absorb water and nutrients. Drought alleviates organic carbon decomposition more than plant carbon input and then decreases the loss of soil carbon (Zhou et al., 2016), which was probably the reason that total organic carbon content was unaffected by drought in this research. Meanwhile, drought dries the fast leaching channels and limits DOC leaching into the deeper soil (Zhang et al., 2019), which causes the accumulation of DOC in the drought conditions. Deng et al. (2021) found that mineral N concentrations (NH_4_^+^‐N and NO_3_^−^‐N) increased under drought condition, which might be because the decrease in root growth attenuated the absorption of mineral N, which was different from this study in terms of NH_4_^+^‐N. The content of NH_4_^+^‐N would grow as the intensity of the drought increased. Ammonification and nitrification with microorganisms' participation determine soil N availability (Beeckman et al., 2018). Drought limits the microbial activity and biomass (Ren et al., 2017), and afterwards has an impact on how much bioavailable nitrogen is produced and transported in the soil (Wang, Li, et al., 2021) and the accumulation of inorganic N in the soil (Wu et al., 2012). Na et al. (2019) found that plant communities utilized a particular N access method to reduce competition for N resources with soil microbes and to limit N loss during drought stress. During drought, Luo et al. (2015) found no correlation between plant phosphorus uptake and soil phosphorus availability, but total phosphorus in soil might rise as a result of arid conditions (Delgado‐Baquerizo et al., 2013), agreeing with the results presented in this study.

### 4.3 The relationship between environmental factors and plant, microbial biomass, and soil nutrients

According to previous studies, the grassland's functional group biomass and overall biomass increased exponentially throughout the MAP gradient, and higher rainfall can lead to greater ANPP (Heisler‐White et al., 2008; Ma et al., 2022). These findings were consistent with our results that the most of important plant indicators showed a positive connection with MAP and drought intensity. Furthermore, in response to exogenous changes of annual precipitation, plants modify their physical features (phenotypic plasticity) (Nicotra et al., 2010), such as decreasing their leaf area as MAP decreased (Navarro et al., 2010) and under drier conditions (Yan et al., 2012). And when available water was limited, leaf growth and extension reduced, resulting in smaller, more compact cells with less intercellular space (Poorter et al., 2009). Consequently, plant biomass or productivity decreases. Soil microbial plays a fundamental role in the regulation of nutrient availability in climate changes. For example, drought can reduce microbial growth, increase microbial mortality, and shift in the composition of active microbial communities along with low mineralization (i.e., the release of inorganic N as NH_4_^+^ and NO_3_^−^ into the environment) (Blagodatskaya, 2013; Mooshammer et al., 2014). Reduced soil water content will change nitrate (dominates inorganic N) mobility, reduce the transfer of nitrate to deeper soil layers and the diffusion of nitrate to plants and microorganisms, and result in accumulation (Evans & Burke, 2013). Moreover, some studies found that inorganic N intake became more weakly than microbial N mineralization and nitrification as temperature increased, resulting in a net increase in inorganic N in soils (Larsen et al., 2011; Niboyet et al., 2011). Our results also indicated that MAT and drought intensity were significantly positively correlated with inorganic N. Soil microbial C and N cycling is strongly influenced by environmental factors. Previous studies revealed that drought increased microbial C:N ratios in grasslands, and indicated that drought may have stronger effects on microbial N than C cycling (Jensen et al., 2003; Zeglin et al., 2013). SR as microbes and plant roots are the performers of the heterotrophic and autotrophic respiration, respectively, drought also have an impact (Li, Qian, et al., 2020; Li, Zhang, et al., 2020). Some studies reported SR has positively correlated with soil temperature or moisture (Liu et al., 2016; Yu et al., 2017), which was consistent with our findings. Probably, increasing in temperature or moisture availability might reduce temperature or water restriction on microbial activities, and hence increased SR (Ru et al., 2018).

## 5 CONCLUSION

Drought had remarkable effects on plant productivity, soil nutrients, and microbial biomass in grassland ecosystems. Drought led to significant decrease in AGB, BGB, ANPP, BNPP, MBC, MBN, and SR. However, some soil nutrients (DOC, TN, NO_3_^−^‐N, and TP) had significant increase under drought stress. These changes were closely correlated with the drought‐related environmental factors (MAT and MAP) affecting the intensity of drought stress. While MAT and altitude were negatively correlated with AGB, height, BNPP, MBC, and MBN, they were positively correlated with MAP. Foreseeable increases in drought intensity in the future would have further negative effect on plant productivity and soil microbial activity of grassland ecosystems, therefore, we should take positive steps to address the negative effects of drought on grassland ecosystems due to climate change.

## AUTHOR CONTRIBUTIONS

**Cheng Liu:** Data curation (equal); formal analysis (equal); methodology (equal); writing – original draft (equal). **Muji Siri:** Data curation (equal); formal analysis (equal); methodology (equal); writing – original draft (equal). **Hui Li:** Data curation (equal); formal analysis (equal); methodology (equal); writing – original draft (equal). **Cheng Ren:** Data curation (equal); formal analysis (equal); methodology (equal); writing – original draft (equal). **Jing Huang:** Data curation (equal); formal analysis (equal); methodology (equal); writing – original draft (equal). **Changliang Feng:** Data curation (equal); formal analysis (equal); methodology (equal); writing – original draft (equal). **Kesi Liu:** Conceptualization (equal); funding acquisition (equal); methodology (equal); supervision (equal); validation (equal); writing – review and editing (equal).

## CONFLICT OF INTEREST STATEMENT

The authors declare that they have no known competing financial interests or personal relationships that could have appeared to influence the work reported in this paper.

## Supporting information


Appendix S1.

## Data Availability

Data available from the Dryad: https://doi.org/10.5061/dryad.wh70rxwsr (Cheng et al., 2023).
