# Supplementary material for: Drought is threatening plant growth and soil nutrients of grassland ecosystems: A meta‐analysis
Source: Ecol Evol. 2023 May 24;13(5):e10092. doi: 10.1002/ece3.10092 (PMC10208897; doi:10.1002/ece3.10092)
Supplement: Supplementary file 1 — Appendix S1. [file ECE3-13-e10092-s001.docx]

**Data sources**

1. Andresen, L. C., Michelsen, A., Jonasson, S., Beier, C., & Ambus, P. (2009). Glycine uptake in heath plants and soil microbes responds to elevated temperature, CO2 and drought. Acta Oecologica, 35(6), 786–796. https://doi.org/10/fc5qsw

2. Canarini, A., Mariotte, P., Ingram, L., Merchant, A., & Dijkstra, F. A. (2018). Mineral-Associated Soil Carbon is Resistant to Drought but Sensitive to Legumes and Microbial Biomass in an Australian Grassland. Ecosystems, 21(2), 349–359. https://doi.org/10/grsxrg

3. Carbonell-Silletta, L., Cavallaro, A., Pereyra, D. A., Askenazi, J. O., Goldstein, G., Scholz, F. G., & Bucci, S. J. (2021). Soil Respiration and N-Mineralization Processes in the Patagonian Steppe Are More Responsive to Nutrient Than to Water Addition [Preprint]. In Review. https://doi.org/10.21203/rs.3.rs-928193/v1

4. Chen, H., Zhao, X., Lin, Q., Li, G., & Kong, W. (2019). Using a combination of PLFA and DNA-based sequencing analyses to detect shifts in the soil microbial community composition after a simulated spring precipitation in a semi-arid grassland in China. Science of The Total Environment, 657, 1237–1245. https://doi.org/10/gqb6dz

5. Dai, X., Wang, P., Liu, R., Hao, Y., & Ji, B. (2020). Effects of extreme drought conditions on arbuscular mycorrhizal fungi community composition in different growing seasons of typical steppe in Inner Mongolia. Prataculture Science, 37(08), 1440–1447.

6. Du, Z., An, H., Wang, B., Wen, Z., Zhang, Y., Wu, X., & Li, Q. (2020). Effects of nutrient addition and precipitation changes on species diversity and biomass of plant communities in desert steppe. Journal of Grassland Science, 28(04), 1100–1110.

7. Evans, S. E., & Burke, I. C. (2013). Carbon and Nitrogen Decoupling Under an 11-Year Drought in the Shortgrass Stepp. Ecosystems., 16(1), 20–33. https://doi.org/10/f4mq6n

8. Fu, Y., Tian, D., Niu, S., & Zhao, K. (2020). Effects of nitrogen and phosphorus supplementation and drought on leaf stoichiometry of dominant plants in an alpine meadow. Journal of Beijing Forestry University, 42(05), 115–123.

9. Fuchslueger, L., Wild, B., Mooshammer, M., Takriti, M., Kienzl, S., Knoltsch, A., Hofhansl, F., Bahn, M., & Richter, A. (2019). Microbial carbon and nitrogen cycling responses to drought and temperature in differently managed mountain grasslands. Soil Biology and Biochemistry, 135, 144–153. https://doi.org/10/gjc4mr

10. Gleixner, G. (2018). Drought-Induced Accumulation of Root Exudates Supports Post-drought Recovery of Microbes in Mountain Grassland. Frontiers in Plant Science, 9.

11. Guo, N., Jiang, J., Wang, G., & Jiao, F. (2020). Effects of different precipitation gradients on the stoichiometric characteristics of grassland communities in loess hilly area. Soil and Water Conservation Bulletin, 40(02), 1–8. https://doi.org/10/grsxqf

12. Guo, X., Zuo, X., Yue, P., Li, X., & Hu, Y. (2021, February 17). Direct and Indirect Effects of Precipitation Change and Nutrients Addition on Desert Steppe Productivity in Inner Mongolia, Northern China. https://doi.org/10.21203/rs.3.rs-244027/v1

13. Guo, X., Zuo, X., Yue, P., Li, X., Hu, Y., Chen, M., & Yu, Q. (2022). Direct and indirect effects of precipitation change and nutrients addition on desert steppe productivity in Inner Mongolia, northern China. Plant and Soil, 471(1–2), 527–540. https://doi.org/10/gn5dd9

14. Huang, Q., Jiao, F., Huang, Y., Li, N., Wang, B., Gao, H., & An, S. (2021). Response of soil fungal community composition and functions on the alteration of precipitation in the grassland of Loess Plateau. Science of The Total Environment, 751, 142273. https://doi.org/10/gn5kb5

15. Jaman, Md. S., Wu, H., Yu, Q., Tan, Q., Zhang, Y., Dam, Q. K., Muraina, T. O., Xu, C., Jing, M., Jia, X., Wang, J., He, N., Luo, W., Knapp, A., Wilkins, K., Collins, S. L., & Luo, Y. (2022). Contrasting responses of plant above and belowground biomass carbon pools to extreme drought in six grasslands spanning an aridity gradient. Plant and Soil, 473(1–2), 167–180. https://doi.org/10/jqfs

16. Jensen, K. D., Beier, C., Michelsen, A., & Emmett, B. (2003). Effects of experimental drought on microbial processes in two temperate heathlands at contrasting water conditions. Applied Soil Ecology. https://doi.org/10/b7tvgd

17. Jia, M., Gao, Z., Gu, H., Zhao, C., Liu, M., Liu, F., Xie, L., Wang, L., Zhang, G., Liu, Y., & Han, G. (2021). Effects of precipitation change and nitrogen addition on the composition, diversity, and molecular ecological network of soil bacterial communities in a desert steppe. PLOS ONE, 16(3), e0248194. https://doi.org/10/grsxq7

18. Jung, C. G., Xu, X., Niu, S., Liang, J., Chen, X., Shi, Z., Jiang, L., & Luo, Y. (2019). Experimental warming amplified opposite impacts of drought vs. Wet extremes on ecosystem carbon cycle in a tallgrass prairie. Agricultural and Forest Meteorology, 276–277, 107635. https://doi.org/10/grsxjr

19. Li, J., Benti, G., Wang, D., Yang, Z., & Xiao, R. (2022). Effect of Alteration in Precipitation Amount on Soil Microbial Community in a Semi-Arid Grassland. Frontiers in Microbiology, 13, 842446. https://doi.org/10/gqm6rj

20. Li, L., Qian, R., Wang, W., Kang, X., Ran, Q., Zheng, Z., Zhang, B., Xu, C., Che, R., Dong, J., Xu, Z., Cui, X., Hao, Y., & Wang, Y. (2020). The intra- and inter-annual responses of soil respiration to climate extremes in a semiarid grassland. Geoderma, 378, 114629. https://doi.org/10/gg7hq2

21. Li, L., Wang, Y., Hu, Z., Li, Y., Shen, Y., Yu, Q., Huang, J., & Wang, C. (2020). Response of soil carbon/nitrogen mineralization potential and soil microbial water sensitivity to extreme drought in meadow steppe. Journal of Applied Ecology, 31(03), 814–820. https://doi.org/10/gm4czx

22. Li, L., Zheng, Z., Biederman, J. A., Qian, R., Ran, Q., Zhang, B., Xu, C., Wang, F., Zhou, S., Che, R., Dong, J., Xu, Z., Cui, X., Hao, Y., & Wang, Y. (2021). Drought and heat wave impacts on grassland carbon cycling across hierarchical levels. Plant, Cell & Environment, 44(7), 2402–2413. https://doi.org/10/gj37pg

23. Li, N., Wang, B., An, S., Jiao, F., & Huang, Q. (2020). Response of soil bacterial community structure to precipitation in grassland on Loess Plateau. Environmental Science, 41(09), 4284–4293. https://doi.org/10/grsxrz

24. Li, X., Han, G., Eller, F., Hui, D., Zhu, L., Chen, L., Chu, X., Song, W., & Xu, J. (2021). Acclimation of coastal wetland vegetation to salinization results in the asymmetric response of soil respiration along an experimental precipitation gradient. Agricultural and Forest Meteorology, 310, 108626. https://doi.org/10/gp9x3b

25. Li, Y., Yu, H., Wang, P., Niu, Y., Fan, J., Zhu, W., & Huang, J. (2020). Effects of precipitation on plant community diversity and C∶N∶P ecological stoichiometry in desert steppe. Chinese Journal of Grassland, 42(01), 117–126. https://doi.org/10/grsxq8

26. Li, Y., Zhou, Z., Lei, L., Ru, J., Song, J., Zhong, M., Tian, R., Zhang, A., Zheng, M., Hui, D., & Wan, S. (2020). Asymmetric responses of soil respiration in three temperate steppes along a T precipitation gradient in northern China revealed by soil-monolith transplanting experiment. Agricultural and Forest Meteorology. https://doi.org/10/grsxjj

27. Li, Z., Peng, Q., Dong, Y., He, Y., Yan, Z., Guo, Y., Qin, S., & Qi, Y. (2022). Response of soil respiration to water and nitrogen addition and its influencing factors: A four-year field experiment in a temperate steppe. Plant and Soil, 471(1–2), 427–442. https://doi.org/10/gnps2w

28. Ma, W., Liang, X., Wang, Z., Luo, W., Yu, Q., & Han, X. (2022). Resistance of steppe communities to extreme drought in northeast China. Plant and Soil, 473(1–2), 181–194. https://doi.org/10/grsxrp

29. Magandana, T. P., Hassen, A., & Tesfamariam, E. H. (2020). Seasonal Herbaceous Structure and Biomass Production Response to Rainfall Reduction and Resting Period in the Semi-Arid Grassland Area of South Africa. Agronomy, 10(11), 1807. https://doi.org/10/grsxr6

30. Mariotte, P., Cresswell, T., Johansen, M. P., Harrison, J. J., Keitel, C., & Dijkstra, F. A. (2020). Plant uptake of nitrogen and phosphorus among grassland species affected by drought along a soil available phosphorus gradient. Plant and Soil, 448(1–2), 121–132. https://doi.org/10/grsxrj

31. Meng, B., Ochoa-Hueso, R., Li, J., Zhong, S., Yao, Y., Yang, X., Collins, S. L., & Sun, W. (2021). Nonlinear decoupling of autotrophic and heterotrophic soil respiration in response to drought duration and N addition in a meadow steppe. Biology and Fertility of Soils, 57(2), 281–291. https://doi.org/10/gmnvk8

32. Na, X., Yu, H., Wang, P., Zhu, W., Niu, Y., & Huang, J. (2019). Vegetation biomass and soil moisture coregulate bacterial community succession under altered precipitation regimes in a desert steppe in northwestern China. Soil Biology and Biochemistry, 136, 107520. https://doi.org/10/gmmxx8

33. Qian, R., Hao, Y., Li, L., Zheng, Z., Wen, F., Cui, X., Wang, Y., Zhao, T., Tang, Z., Du, J., & Xue, K. (2022). Joint control of seasonal timing and plant function types on drought responses of soil respiration in a semiarid grassland. Frontiers in Plant Science, 13, 974418. https://doi.org/10/grsxsq

34. Sardans, J. (2008). Changes in soil enzymes related to C and N cycle and in soil C and N content under prolonged warming and drought in a Mediterranean shrubland. Applied Soil Ecology. https://doi.org/10/dxh897

35. Slette, I. J., Hoover, D. L., Smith, M. D., & Knapp, A. K. (2023). Repeated extreme droughts decrease root production, but not the potential for post-drought recovery of root production, in a mesic grassland. Oikos, 2023(1), e08899. https://doi.org/10/jqfq

36. Song, X., Wang, Y., Wang, Z., Kang, H., Liu, C., Li, Z., Qu, Z., Han, G., & Wang, Z. (2019). Relationship between soil respiration and subsurface biomass in desert steppe under different grazing intensities and water treatments. Journal of Grassland Science, 27(04), 962–968.

37. Sun, Y., Tian, Q., Lv, P., Guo, A., Zhang, S., & Zuo, X. (2020). Effects of extreme drought and nitrogen addition on species diversity, leaf traits and productivity in semi-arid sandy grasslands. Arid Zone Research, 37(06), 1569–1579. https://doi.org/10/grsxqs

38. Wang, F., Li, Z., Su, F., Guo, H., Wang, P., Guo, J., Zhu, W., Wang, Y., & Hu, S. (2022). Sensitive Groups of Bacteria Dictate Microbial Functional Responses to Short-term Warming and N Input in a Semiarid Grassland. Ecosystems, 25(6), 1346–1357. https://doi.org/10/gn5c88

39. Wang, J., Zhang, L., Zhao, R., & Xie, Z. (2020). Responses of different life types to precipitation changes in desert steppe. Journal of Applied Ecology, 31(03), 778–786. https://doi.org/10/gmsmfr

40. Wang, N., Li, L., Dannenmann, M., Luo, Y., Xu, X., Zhang, B., Chen, S., Dong, K., Huang, J., Xu, X., & Wang, C. (2021). Seasonality of gross ammonification and nitrification altered by precipitation in a semi-arid grassland of Northern China. Soil Biology and Biochemistry, 154, 108146. https://doi.org/10/gmmxzb

41. Wang, Y., Xie, Y., Ma, H., Zhang, Y., Zhang, J., Zhang, H., Luo, X., & Li, J. (2022). Responses of Soil Microbial Communities and Networks to Precipitation Change in a Typical Steppe Ecosystem of the Loess Plateau. Microorganisms, 10(4), 817. https://doi.org/10/gqm6sz

42. Wang, Z., Mckenna, T. P., Schellenberg, M. P., Tang, S., Zhang, Y., Ta, N., Na, R., & Wang, H. (2019). Soil respiration response to alterations in precipitation and nitrogen addition in a desert steppe in northern China. Science of The Total Environment, 688, 231–242. https://doi.org/10/grsxsd

43. Xu, X., Liu, H., Wang, W., Hu, G., Wu, X., & Song, Z. (2021). Effects of manipulated precipitation on aboveground net primary productivity of grassland fields: Controlled rainfall experiments in Inner Mongolia, China. Land Degradation & Development, 32(5), 1981–1992. https://doi.org/10/grsxjp

44. Yan, Y., Wang, J., Tian, D., Luo, Y., Xue, X., Peng, F., He, J.-S., Liu, L., Jiang, L., Wang, X., Wang, Y., Song, L., & Niu, S. (2022). Sustained increases in soil respiration accompany increased carbon input under long-term warming across global grasslands. Geoderma, 428, 116157. https://doi.org/10/grsxsr

45. Yang, X., Zhu, K., Loik, M. E., & Sun, W. (2021). Differential responses of soil bacteria and fungi to altered precipitation in a meadow steppe. Geoderma, 384, 114812. https://doi.org/10/gpwz77

46. Ye, R., Liu, G., Chang, H., Shan, Y., Mu, L., Wen, C., Te, R., Wu, N., Shi, L., Liu, Y., Wang, H., Yun, X., Liu, G., & Li, F. (2020). Response of plant traits of Stipa breviflora to grazing intensity and fluctuation in annual precipitation in a desert steppe, northern China. Global Ecology and Conservation, 24, e01237. https://doi.org/10/grmkbz

47. Zhang, J., Ru, J., Song, J., Li, H., Li, X., Ma, Y., Li, Z., Hao, Y., Chi, Z., Hui, D., & Wan, S. (2022). Increased precipitation and nitrogen addition accelerate the temporal increase in soil respiration during 8‐year old‐field grassland succession. Global Change Biology, 28(12), 3944–3959. https://doi.org/10/gpn3dd

48. Zhang, J., Shen, X., Mu, B., Shi, Y., Yang, Y., Wu, X., Mu, C., & Wang, J. (2021). Moderately prolonged dry intervals between precipitation events promote production in Leymus chinensis in a semi-arid grassland of Northeast China. BMC Plant Biology, 21(1), 147. https://doi.org/10/grsxrh

49. Zhang, J., Zuo, X., Zhao, X., Ma, J., & Medina-Roldán, E. (2020). Effects of rainfall manipulation and nitrogen addition on plant biomass allocation in a semiarid sandy grassland. Scientific Reports, 10(1), 9026. https://doi.org/10/gj37pz

50. Zhang, K., Shi, Y., Jing, X., He, J.-S., Sun, R., Yang, Y., Shade, A., & Chu, H. (2016). Effects of Short-Term Warming and Altered Precipitation on Soil Microbial Communities in Alpine Grassland of the Tibetan Plateau. Frontiers in Microbiology, 7. https://doi.org/10/grsxrc

51. Zhang, L., Xie, Z., Zhao, R., & Zhang, Y. (2018). Plant, microbial community and soil property responses to an experimental precipitation gradient in a desert grassland. Applied Soil Ecology, 127, 87–95. https://doi.org/10/gdgqkr

52. Zhang, R., Zhao, X., Wang, S., Zuo, X., & wang, R. (2019). Effects of extreme drought on species diversity and aboveground biomass carbon and nitrogen in desert steppe communities. Journal of Ecology and Environment, 28(04), 715–722. https://doi.org/10/grsxqb

53. Zhang, R., Zhao, X., Zuo, X., Degen, A. A., Li, Y., Liu, X., Luo, Y., Qu, H., Lian, J., & Wang, R. (2020). Drought-induced shift from a carbon sink to a carbon source in the grasslands of Inner Mongolia, China. CATENA, 195, 104845. https://doi.org/10/gnhtsq

54. Zhang, R., Zhao, X., Zuo, X., Liu, X., Qu, H., Ma, X., Liu, L., Chen, J., & Liu, liping. (2019). Response of Stipa glareosa community species diversity and aboveground biomass to rainfall in desert steppe. Chinese Desert, 39(02), 45–52.

55. Zhang, Y. (2020). Effects of extreme drought on primary productivity and biomass of underground root systems in Inner Mongolia steppe [PhD Thesis]. Chinese Academy of Agricultural Sciences.

56. Zhou, Y., Zhang, Y., Ma, W., Liang, X., Ma, X., & Wang, Z. (2020). Effects of nitrogen addition and drought on five plant traits in Hulunbuir Grassland. Journal of Ecological Environment, 29(01), 41–48. https://doi.org/10/grsxq3

57. Zhu, E., Cao, Z., Jia, J., Liu, C., Zhang, Z., Wang, H., Dai, G., He, J., & Feng, X. (2021). Inactive and inefficient: Warming and drought effect on microbial carbon processing in alpine grassland at depth. Global Change Biology, 27(10), 2241–2253. https://doi.org/10/gh3qpr

58. Zhu, G., Guo, N., Han, Y., Lv, G., Wang, Z., & Wang, C. (2021). Effects of extreme drought on plant community species diversity and soil properties in desert steppe of Inner Mongolia. Chinese Journal of Grassland, 43(03), 52–59. https://doi.org/10/gp3b64

59. Zhu, W., Xu, Y., Wang, P., Yu, H., & Huang, J. (2020). Effects of precipitation and N addition on C∶N∶P ecological stoichiometric characteristics of plants and soil microorganisms in desert steppe. Journal of Northwestern Botany, 40(04), 676–687.

60. Zuo, X., Li, X., Yue, P., Guo, A., Yue, X., Xu, C., Knapp, A. K., Smith, M. D., Luo, W., Allington, G. R. H., & Yu, Q. (2022). Drought-driven shifts in relationships between plant biodiversity and productivity in temperate steppes. Functional Ecology, 36(12), 2917–2928. <https://doi.org/10/grsxsm>

61. Arfin Khan, M.A.S., Grant, K., Beierkuhnlein, C., Kreyling, J. and Jentsch, A. (2014) ‘Climatic extremes lead to species-specific legume facilitation in an experimental temperate grassland’, Plant and Soil, 379(1), pp. 161–175. Available at: https://doi.org/10/gr5dkt.

62. Broderick, C.M., Wilkins, K., Smith, M.D. and Blair, J.M. (2022) ‘Climate legacies determine grassland responses to future rainfall regimes’, Global Change Biology, 28(8), pp. 2639–2656. Available at: https://doi.org/10/gqfnfg.

63. Canarini, A., Carrillo, Y., Mariotte, P., Ingram, L. and Dijkstra, F.A. (2016) ‘Soil microbial community resistance to drought and links to C stabilization in an Australian grassland’, Soil Biology and Biochemistry, 103, pp. 171–180. Available at: https://doi.org/10/f9dcmb.

64. Canarini, A., Mariotte, P., Ingram, L., Merchant, A. and Dijkstra, F.A. (2018) ‘Mineral-Associated Soil Carbon is Resistant to Drought but Sensitive to Legumes and Microbial Biomass in an Australian Grassland’, Ecosystems, 21(2), pp. 349–359. Available at: https://doi.org/10/grsxrg.

65. Cantarel, A.A.M., Bloor, J.M.G. and Soussana, J.-F. (2013) ‘Four years of simulated climate change reduces above-ground productivity and alters functional diversity in a grassland ecosystem’, Journal of Vegetation Science, 24(1), pp. 113–126. Available at: https://doi.org/10/f4gn7q.

66. Domínguez, M.T., Holthof, E., Smith, A.R., Koller, E. and Emmett, B.A. (2017) ‘Contrasting response of summer soil respiration and enzyme activities to long-term warming and drought in a wet shrubland (NE Wales, UK)’, Applied Soil Ecology, 110, pp. 151–155. Available at: https://doi.org/10.1016/j.apsoil.2016.11.003.

67. Haugwitz, M.S., Bergmark, L., Priemé, A., Christensen, S., Beier, C. and Michelsen, A. (2014) ‘Soil microorganisms respond to five years of climate change manipulations and elevated atmospheric CO2 in a temperate heath ecosystem’, Plant and Soil, 374(1), pp. 211–222. Available at: https://doi.org/10/f5mg57.

68. Hofer, D., Suter, M., Buchmann, N. and Lüscher, A. (2017) ‘Nitrogen status of functionally different forage species explains resistance to severe drought and post-drought overcompensation’, Agriculture, Ecosystems & Environment, 236, pp. 312–322. Available at: https://doi.org/10/f9p2cz.

69. Hoover, D.L., Hajek, O.L., Smith, M.D., Wilkins, K., Slette, I.J. and Knapp, A.K. (2022) ‘Compound hydroclimatic extremes in a semi-arid grassland: Drought, deluge, and the carbon cycle’, Global Change Biology, 28(8), pp. 2611–2621. Available at: https://doi.org/10/gpdf6t.

70. Hoover, D.L., Knapp, A.K. and Smith, M.D. (2014) ‘Resistance and resilience of a grassland ecosystem to climate extremes’, Ecology, 95(9), pp. 2646–2656. Available at: https://doi.org/10/f6jr7q.

71. Khalili, B., Ogunseitan, O.A., Goulden, M.L. and Allison, S.D. (2016) ‘Interactive effects of precipitation manipulation and nitrogen addition on soil properties in California grassland and shrubland’, Applied Soil Ecology, 107, pp. 144–153. Available at: https://doi.org/10/f87dkj.

72. Knapp, A.K., Chen, A., Griffin-Nolan, R.J., Baur, L.E., Carroll, C.J.W., Gray, J.E., Hoffman, A.M., Li, X., Post, A.K., Slette, I.J., Collins, S.L., Luo, Y. and Smith, M.D. (2020) ‘Resolving the Dust Bowl paradox of grassland responses to extreme drought’, Proceedings of the National Academy of Sciences, 117(36), pp. 22249–22255. Available at: https://doi.org/10/gk83b5.

73. Munson, S.M., Bradford, J.B., Butterfield, B.J. and Gremer, J.R. (2022) ‘Primary production responses to extreme changes in North American Monsoon precipitation vary by elevation and plant functional composition through time’, Journal of Ecology, 110(9), pp. 2232–2245. Available at: https://doi.org/10/gr48dk.

74. Niboyet, A., Bardoux, G., Barot, S. and Bloor, J.M.G. (2017) ‘Elevated CO2 mediates the short-term drought recovery of ecosystem function in low-diversity grassland systems’, Plant and Soil, 420(1), pp. 289–302. Available at: https://doi.org/10/gcn65b.

75. Schaeffer, S.M., Homyak, P.M., Boot, C.M., Roux-Michollet, D. and Schimel, J.P. (2017) ‘Soil carbon and nitrogen dynamics throughout the summer drought in a California annual grassland’, Soil Biology and Biochemistry, 115, pp. 54–62. Available at: https://doi.org/10/gck677.

76. Slette, I.J., Blair, J.M., Fay, P.A., Smith, M.D. and Knapp, A.K. (2022) ‘Effects of Compounded Precipitation Pattern Intensification and Drought Occur Belowground in a Mesic Grassland’, Ecosystems, 25(6), pp. 1265–1278. Available at: https://doi.org/10/gn5tgp.

77. Slette, I.J., Hoover, D.L., Smith, M.D. and Knapp, A.K. (2023) ‘Repeated extreme droughts decrease root production, but not the potential for post-drought recovery of root production, in a mesic grassland’, Oikos, 2023(1), p. e08899. Available at: https://doi.org/10/jqfq.

78. de Vries, F.T., Williams, A., Stringer, F., Willcocks, R., McEwing, R., Langridge, H. and Straathof, A.L. (2019) ‘Changes in root-exudate-induced respiration reveal a novel mechanism through which drought affects ecosystem carbon cycling’, New Phytologist, 224(1), pp. 132–145. Available at: https://doi.org/10/gjvhdv.

79. Wilcox, K.R., von Fischer, J.C., Muscha, J.M., Petersen, M.K. and Knapp, A.K. (2015) ‘Contrasting above- and belowground sensitivity of three Great Plains grasslands to altered rainfall regimes’, Global Change Biology, 21(1), pp. 335–344. Available at: https://doi.org/10/f6t44v.

80. Zaré, A., Bougma, C., Karim, O. and Ouédraogo, O. (2022) ‘Assessing the competitive ability of the invader Senna obtusifolia with coexisting natives species under different water stress regimes’, Journal of Experimental Biology and Agricultural Sciences [Preprint]. Available at: https://doi.org/10/gr48hb.
